# A Novel Fault-Identification Method for Micro Coils of EMECs Based on a Composite Analytical Model Combining a 2D Thermal Model and a 1D-CNN

**DOI:** 10.3390/mi17070777

**Published:** 2026-06-26

**Authors:** Aobo Wang, Jiaxin You, Xu Tan, Yutong Xue, Xinyu Jin

**Affiliations:** 1 Institute of Reliability in Electrical Apparatus and Electronics, Harbin Institute of Technology, Harbin 150001, China; 23s106190@stu.hit.edu.cn (A.W.); youjiaxin@hit.edu.cn (J.Y.); xueyutong@hityg.com.cn (Y.X.); jinxinyu@hityg.com.cn (X.J.); 2 Shaanxi Qunli Electrician Co., Ltd., Baoji 721300, China

**Keywords:** coil fault, thermal circuit, two-dimensional analytical model, reusable thermal resistance, 1D-CNN

## Abstract

This paper proposes a novel fault-identification method for micro-coils in relays with forcibly guided contacts, a type of electromechanical elementary component (EMEC), combining a composite analytical model, a 2D thermal model, and a 1D-CNN. A low-order thermal circuit with one central node and four boundary nodes is established, while a two-dimensional anisotropic Poisson equation is used as a high-order calibration model. The two models are coupled through iterative correction of reusable thermal resistances. For thermal aging, enamel-film delamination, and inter-turn short-circuit faults, thermal-conductivity attenuation, asymmetric branch-resistance perturbation, and localized abnormal heat-source injection are introduced to generate physically constrained temperature sequences. Orthogonal centerline temperature distributions are extracted as one-dimensional feature vectors for 1D-CNN classification. Simulation results show that the hybrid model has an error of approximately 1.7% compared with finite-element results, and the trained 1D-CNN achieves 98.13% accuracy on 160 test samples. Experimental reconstruction and deep-feature visualization further verify its ability to distinguish normal, aging, delamination, and local short-circuit states.

## 1. Introduction

Electromechanical elementary components (EMECs) refer to compact functional units that realize electrical-to-mechanical conversion in industrial and safety systems. Although small in size, these components integrate electromagnetic, mechanical, and thermal functions within highly compact internal structures. In this study, relay micro coils are considered representative electromechanical elementary components, because their effective thermal behavior is governed by millimeter-scale winding geometry and limited internal heat-dissipation paths. Although small, their internal structures integrate electromagnetic, mechanical, and thermal functions within highly compact spaces. For example, a relay’s effective thermal behavior is governed by millimeter-scale winding geometry and limited internal heat-dissipation paths. This high-integration characteristic makes local degradation difficult to observe directly. However, once thermal accumulation or insulation deterioration occurs, the entire component’s reliability is compromised. Therefore, fault identification requires methods capturing internal physical-state variations under compact structural constraints.

Among these components, relays with forcibly guided contacts are vital safety devices due to their mechanically linked contacts. Existing relay studies have explored contact-state variation and electrothermal behavior [1], equivalent heat-source modeling [2], magnetic-material selection [3], and data-driven contact-resistance prediction [4]. However, these studies mainly focus on contact-side degradation or overall operating states, leaving the fault identification of the internal micro coil insufficiently investigated.

From the perspective of coil faults, inter-turn short-circuit studies in electrical machines show that localized winding faults produce identifiable electromagnetic and thermal responses [5]. Online diagnosis [6], fault-tolerant control [7], search-coil-based localization [8], and real-time signal extraction [9] demonstrate the feasibility of early fault detection. Physical-model-based sample construction also enables mechanism-constrained data generation [10]. These studies provide useful references, but their objects are mostly rotating machines and their diagnostic features are generally electromagnetic signals, rather than the spatial temperature sequences of compact relay coils.

Thermal-circuit and hybrid modeling provide an important route for fast, interpretable temperature-field prediction. Lumped-parameter thermal networks are widely used for rapid hotspot estimation [11,12,13], while hybrid models couple low-dimensional networks with analytical fields to improve spatial-temperature representation efficiently [14]. Other models emphasize end-winding heat-transfer [15], distributed winding geometry [16], fill factors [17], and real-time estimation [18]. Nonetheless, these works are mostly developed for motors or transformers with regular structures. They are not directly applicable to relay coils featuring compact geometry, limited measurement points, asymmetric boundaries, and local thermal distortion caused by faults.

Finally, intelligent diagnosis methods can identify fault patterns from temperature fields. Research shows that machine learning and deep CNNs can effectively extract discriminative features from multi-stress monitoring signals [19,20,21,22]. Furthermore, thermal-image classification confirms that thermal-field patterns provide high-value information for distinguishing fault states [23,24], supporting embedded AI diagnosis [25]. Therefore, this paper proposes a relay coil fault-identification method integrating a composite thermal circuit, a two-dimensional analytical thermal model, and a 1D-CNN. Compared with pure finite-element models, it supports rapid sample generation; compared with conventional lumped circuits, it preserves local hotspot and boundary asymmetry; and compared with purely data-driven models, it retains the mapping between fault mechanisms and temperature-field distortion.

To clarify the novelty of this work, the proposed method does not simply combine a lumped thermal circuit, two-dimensional thermal-field reconstruction and a 1D-CNN classifier. Its main contribution is a mechanism-constrained framework for relay micro coils, in which reusable thermal-resistance correction links the low-order thermal circuit with boundary-driven two-dimensional reconstruction, and fault-parameter injection generates compact orthogonal temperature sequences for 1D-CNN diagnosis. A comparison with representative studies is provided in Table 1.

This paper proposes a fault-identification method for the micro coil of electromechanical elementary components by integrating a composite thermal circuit, a two-dimensional analytical thermal model, and a one-dimensional convolutional neural network. The representative object is the micro coil in relays with forcibly guided contacts, and typical fault modes including thermal aging, local short/crack, and film delamination are considered. Section 2 establishes the composite thermal-circuit model and the two-dimensional analytical coupling model. A low-order thermal network is first constructed to describe the main heat-transfer paths of the coil, and then a rectangular cross-section analytical model is introduced to reconstruct the spatial temperature field and correct the reusable thermal resistances. Section 3 develops the fault-identification framework based on the hybrid thermal model and 1D-CNN. The electrothermal fault mechanisms are mapped into temperature-field features, which are further vectorized and used for neural-network training and classification. Section 4 presents the experimental validation and steady-state boundary-driven model prediction, including the measurement platform, boundary-condition reconstruction, fault-identification results, and discriminant analysis. Finally, Section 5 summarizes the main conclusions and discusses the effectiveness of the proposed method.

## 2. Composite Thermal Circuit and Two-Dimensional Analytical Coupling Model

### 2.1. Construction of the Basic Composite Thermal-Circuit Model for the Relay Coil

**Figure 1:**
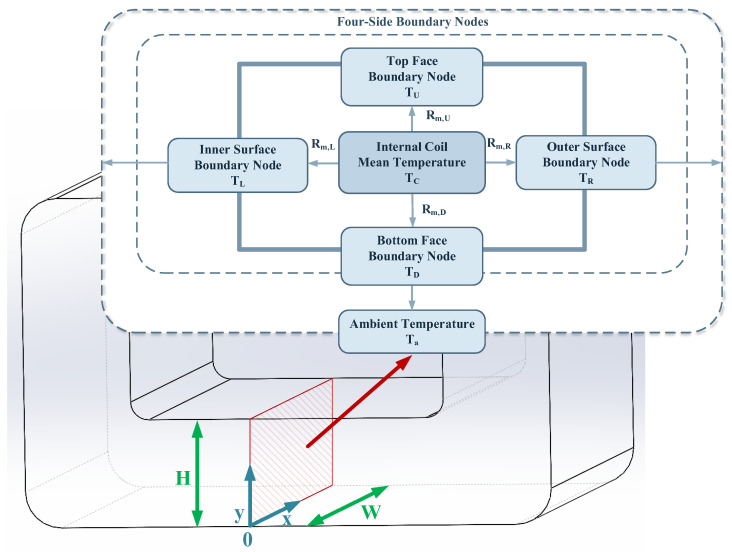
Composite thermal-circuit topology of the relay coil with one central node and four boundary nodes.

The basis for constructing the hybrid thermal model is a lumped-parameter thermal network that can rapidly calculate the global temperature distribution. An electromagnetic coil consists of copper wire, insulation layers, air gaps, and other materials, and thus exhibits pronounced equivalent anisotropy. A single-node thermal model cannot reflect the temperature gradient from the coil interior to the surface. Therefore, according to the geometric characteristics of the rectangular coil cross-section, this paper establishes a composite thermal network based on reusable thermal resistances. The resulting composite thermal-circuit topology is illustrated in Figure 1.

The network introduces a central node *T_c_* representing the internal average temperature of the coil and connects it through reusable thermal resistances in four orthogonal directions to boundary nodes representing the inner surface, outer surface, upper end face, and lower end face of the coil. This topology retains the high computational speed of a low-order thermal network, while using the connection thermal resistances as reusable interfaces carrying two-dimensional temperature-field information. These resistances are dynamically corrected in subsequent hybrid iterations, thereby compensating for the insufficient description of two-dimensional anisotropic heat conduction by a one-dimensional thermal circuit.

In the composite thermal circuit, the branches connecting the central node and boundary nodes mainly act as conductive thermal resistances, which are calculated based on Fourier’s law of heat conduction. For the conductive thermal resistance *R_cd_* of the coil in a specific heat-flow direction, the basic physical expression is as follows:

(1) $$R_{cd}=\frac{L}{\lambda A}$$

where *L* is the characteristic heat-conduction length in this direction, *A* is the cross-sectional area perpendicular to the heat-flow direction, and *λ* is the equivalent thermal conductivity.

In the hybrid model proposed in this paper, the above conductive thermal resistance is defined as the base value of the reusable thermal resistance *R_m_*. In the subsequent iterative correction process, a correction coefficient is introduced on this base value to compensate for the deviation between one-dimensional geometric estimation and the actual two-dimensional heat flow.

For heat exchange among the relay housing, coil surface, and external environment, the model adopts a heat-dissipation thermal resistance. According to Newton’s law of cooling, this heat-dissipation thermal resistance *R_cv_* can be expressed as follows:

(2) $$R_{cv}=\frac{1}{hS}$$

where *S* is the effective surface area participating in convective heat transfer, and *h* is the convective heat-transfer coefficient. Considering that the actual heat-dissipation conditions of a relay may be affected by installation mode, airflow, and housing structure, *h* can be given by empirical formulas and should be further corrected through experimental calibration.

### 2.2. Two-Dimensional Analytical Model for the Rectangular Cross-Section

To correct the limitations of the composite thermal-circuit model in describing complex two-dimensional heat flow, this paper establishes a two-dimensional analytical model based on a rectangular cross-section. Under given boundary temperature conditions, this model reconstructs the continuous temperature distribution *T*(*x*,*y*) inside the coil cross-section and calculates the average and maximum temperatures, which serve as reference targets for iterative identification of the reusable thermal resistance.

The rectangular cross-section perpendicular to the winding direction of the relay coil is selected as the solution domain, and the geometric definition of the rectangular computational cross-section is shown in Figure 1. The width is *W* = 17 mm and the height is *H* = 3 mm, as indicated by the green arrows in the figure. A two-dimensional Cartesian coordinate system (*x*, *y*) is established with the lower-left corner of the rectangular section as the origin, and the x- and y-axes are indicated by the blue arrows in the figure, where *x*∈[0, *W*] and *y*∈[0, *H*]. The red arrow in the figure indicates that the coil cross-section is equivalent to a composite thermal-network topology.

Under steady-state coil operation, assuming that the equivalent material of the section is homogeneous and Joule heat is approximately uniformly distributed under the healthy condition, the steady-state temperature distribution in the section satisfies the two-dimensional anisotropic Poisson equation with a constant internal heat source:

(3) $$\lambda_{x}\frac{\partial^{2}T}{\partial x^{2}}+\lambda_{y}\frac{\partial^{2}T}{\partial y^{2}}=-q_{v}$$

where *T*(*x*,*y*) is the temperature at an arbitrary point in the section; *λ_x_* and *λ_y_* are the equivalent thermal conductivities of the coil composite material in the radial direction (lamination direction) and axial direction (wire-arrangement direction), respectively; and *q_v_* is the volumetric heat-generation rate (W/m^3^), obtained by dividing the total coil power loss by the effective volume.

This equation describes the diffusion of heat from the internal heat source toward the surrounding boundaries within the rectangular domain and provides the mathematical basis for capturing local heat accumulation and temperature-gradient variations.

The solution of the analytical model depends on explicit boundary conditions. In the hybrid modeling strategy, the temperatures of the four boundaries are directly given by the four boundary-node temperatures output by the composite thermal-circuit model.

The four edges of the rectangular cross-section are set to satisfy Dirichlet boundary conditions, which can be mathematically expressed as follows:

(4) $$\left\{\begin{array}{l} T(0,y)=T_{L}\\ T(W,y)=T_{R}\\ T(x,0)=T_{D}\\ T(x,H)=T_{U} \end{array}\right.$$

where *T_L_*, *T_R_*, *T_D_*, and *T_U_* denote the left, right, lower, and upper boundary-node temperatures output by the composite thermal circuit at the current iteration step, respectively. This coupling method ensures that the two-dimensional analytical model always operates under a macroscopic heat-dissipation environment consistent with the low-order thermal circuit.

For the above boundary-value problem, the separation-of-variables method is used. According to the linear superposition principle, the temperature field *T*(*x*,*y*) is decomposed into a general solution *T_h_* determined by the nonuniform boundary temperatures and a particular solution *T_p_* determined by the internal heat source. By constructing eigenfunctions and solving the Fourier-series coefficients, the analytical expression of the temperature field containing series terms can be obtained as follows:

(5) $$T(x,y)=\sum_{n=1}^{\infty }\left[F_{n}(x,T_{b})\sin\left(\frac{n\pi y}{H}\right)+G_{n}(y,T_{b})\sin\left(\frac{n\pi x}{W}\right)\right]+\Psi (x,y,q_{v})$$

where *T_b_* is the boundary-temperature vector, the functions *F_n_* and *G_n_* contain hyperbolic sine (sinh) and hyperbolic cosine (cosh) terms, and *ψ*(*x*,*y*) is the particular-solution function describing the temperature rise caused by the internal heat source. The number of series terms should be determined through truncation-error testing; in the case study of this paper, the first 5–10 terms are sufficient to meet engineering calculation accuracy.

Based on this analytical solution, the theoretical average temperature *T_avg_* of the section can be directly calculated by integration:

(6) $$T_{avg}=\frac{1}{WH}\int_{0}^{H}\int_{0}^{W}T(x,y)dxdy$$

Meanwhile, by solving the gradient equation ∇*T* = 0, the internal hotspot coordinates (*x_max_*, *y_max_*) of the section can be located and the theoretical maximum temperature *T_max_* can be obtained. *T_avg_* and *T_max_* together constitute important outputs for reusable thermal-resistance identification and model-performance evaluation.

### 2.3. Iterative Correction Strategy Based on Reusable Thermal Resistance

The composite thermal circuit constructed in Section 2.1 has the advantage of high computational efficiency, but the initial value of its reusable thermal resistance *R_m_* is estimated only from geometric dimensions and does not yet fully include the two-dimensional heat-accumulation effect inside the rectangular cross-section. The two-dimensional analytical model in Section 2.2 has higher accuracy, but repeated series calculations still introduce computational overhead in large-scale sample generation and edge deployment. Therefore, this paper proposes an iterative correction strategy with closed-loop coupling between the analytical model and the thermal-circuit model.

This strategy uses the two-dimensional analytical model as the reference model under the current boundary conditions and dynamically identifies the reusable thermal resistance in the composite thermal circuit through numerical iteration. The central-node temperature *T_c_* of the composite thermal circuit is made to approach the average temperature *T_avg_* of the analytical model, thereby reusing the main temperature-field information of the two-dimensional model within the low-dimensional thermal circuit.

To couple the one-dimensional node model with the two-dimensional field model, this paper defines the following bidirectional data interaction and error–feedback mechanism.

1. Forward driving: Boundary-condition transfer.

In the kth iteration, the composite thermal circuit uses the current reusable thermal resistance to solve the steady-state temperature distribution and extracts the four boundary-node temperatures {*T_L_*,*T_R_*,*T_D_*,*T_U_*}^(*k*)^ as Dirichlet boundary conditions for the two-dimensional analytical model.

2. Reverse calibration: Error-signal generation.

After receiving the boundary conditions, the two-dimensional analytical model calculates the cross-sectional average temperature *T_avg_* through series summation and integration. *T_avg_* is then compared with the central-node temperature *T_c_* of the composite thermal circuit to obtain the error signal that drives parameter updating.

Based on the above feedback mechanism, a parameter-adaptive iterative algorithm is used to identify the reusable thermal resistance. The specific procedure is as follows: Step 1: Set the initial reusable thermal resistance *R_m_*_,0_, convergence threshold *δ*, and maximum adjustment step *α*. Step 2: Run the composite thermal-circuit model at step k to output the boundary-temperature group and the predicted central-node temperature *T_c_*^(*k*)^. Step 3: Substitute the boundary temperatures into the two-dimensional analytical model to calculate the sectional average temperature *T_avg_*^(*k*)^. Step 4: Calculate the relative error *ε*^(*k*)^ of the composite thermal-circuit prediction with respect to the analytical reference value:

(7) $$\varepsilon^{\left(k\right)}=\frac{T_{c}^{\left(k\right)}-T_{\mathrm{avg}}^{\left(k\right)}}{T_{\mathrm{avg}}^{\left(k\right)}}$$

Step 5: Update the reusable thermal resistance according to the heat–balance relationship of the central node in the composite thermal circuit. Reducing the conductive thermal resistance enhances heat transfer toward the boundaries and lowers the central temperature, whereas increasing the conductive thermal resistance raises the central temperature. Accordingly, a physically consistent proportional updating strategy is adopted.

When *ε*^(*k*)^ > 0, the composite thermal circuit overestimates the central temperature, indicating insufficient equivalent heat-dissipation capability; therefore, the reusable thermal resistance should be reduced to enhance heat conduction.

When *ε*^(*k*)^ < 0, the composite thermal circuit underestimates the central temperature, indicating excessively strong equivalent heat dissipation; therefore, the reusable thermal resistance should be increased to represent the internal thermal-resistance lag effect. The updating formula adopts a proportional adjustment form:

(8) $$R_{m}^{\left(k+1\right)}=R_{m}^{\left(k\right)}\times \left(1+\alpha \cdot \mathrm{sign}\left(\varepsilon^{\left(k\right)}\right)\cdot \left|\varepsilon^{\left(k\right)}\right|\right)$$

Step 6: Determine whether |*ε*^(*k*)^| is smaller than the convergence threshold *δ*. If the condition is satisfied, the iteration is terminated and the reusable thermal resistance is locked; otherwise, let *k* = *k* + 1 and return to Step 2. To avoid over-adjustment, upper and lower limits may be set for the correction coefficient, and the number of iterations and final error are recorded.

This update direction is consistent with the central-node heat balance: when *T_c_*^(*k*)^ > *T_avg_*^(*k*)^, R_m_ is reduced to strengthen equivalent heat conduction, whereas when *T_c_*^(*k*)^ < *T_avg_*^(*k*)^, R_m_ is increased to weaken excessive equivalent heat dissipation. The bounded proportional correction in Equation (9) is used to avoid over-adjustment and improve convergence stability.

After convergence of the reusable thermal resistance, the model not only obtains the calibrated average temperature but also synchronously obtains the hotspot position and maximum temperature from the two-dimensional analytical model, providing a temperature-field basis for subsequent fault-sample generation.

**Figure 2:**
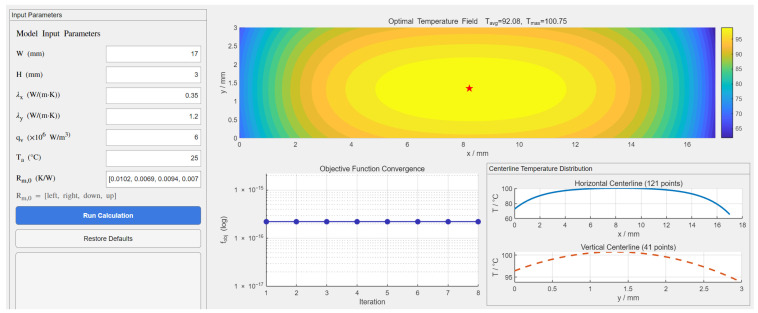
The composite thermal circuit model in mathematical simulation software.

Figure 2 shows the visual parameter input and output interface of the composite thermal-circuit model in mathematical simulation software.

Table 2 lists the main input parameters of the hybrid model proposed in this paper. The red star in the figure represents the hotspot location. It should be noted that the equivalent thermal conductivities, volumetric heat-generation rate, and initial thermal resistance all affect model outputs; in subsequent applications, they should be calibrated using material parameters, power tests, and boundary temperature measurements. The four initial thermal resistance terms in Table 2 denote the baseline conductive thermal resistances of the corresponding heat-transfer branches in the equivalent thermal network before fault-related parameter correction. They are calculated from the initial geometric dimensions and material thermal conductivities, and are used as reference values for subsequent thermal-resistance updating under different fault conditions.

To examine the influence of the reduced-order assumptions, a one-factor sensitivity calculation was performed for the key thermal parameters. The normalized sensitivity coefficient was defined as

(9) $$S_{p}^{Y}=\frac{\left\{\left[Y(p+\Delta p)-Y(p-\Delta p)\right]/(2Y_{0})\right\}}{\Delta p/p_{0}}$$

where Y denotes *T_avg_*, *T_max_*, or the centerline temperature-sequence deviation. Here, *p* is the perturbed parameter, Δ*p* is the perturbation magnitude, *p*_0_ is the baseline value of the parameter, *Y*_0_ is the baseline output, and *Y*(*p* + Δ*p*) and *Y*(*p* − Δ*p*) are the corresponding outputs under positive and negative perturbations, respectively. In Table 3, |*S_T_avg_*|, *|S_T_max_|*, and |*S_s_eq_*| denote the absolute normalized sensitivity coefficients of the average temperature, maximum temperature, and centerline temperature-sequence deviation, respectively.

The results indicate that *q_v_*, *h*, and *R_m_* are the dominant parameters for the overall temperature level, while directional reusable resistance and reconstructed boundary temperatures mainly affect the spatial sequence shape; the equivalent conductivities mainly influence the temperature-gradient distribution.

**Figure 3:**
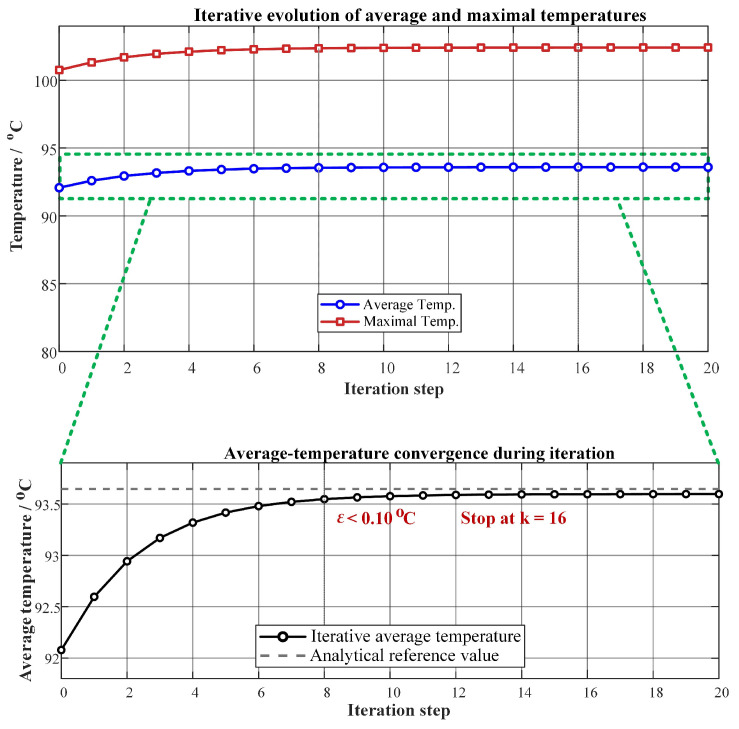
Iterative convergence curves of the maximum and average coil temperatures.

To verify the stability of the iterative calculation process, this paper records the changes in the maximum and average coil temperatures with the number of iterations, as shown in Figure 3.

The iteration curves show that the maximum and average temperatures change rapidly in the initial stage and tend to stabilize after approximately 3–4 iterations, without obvious oscillation or divergence. This indicates that the proposed reusable thermal-resistance correction strategy has good convergence under this operating condition.

**Figure 4:**
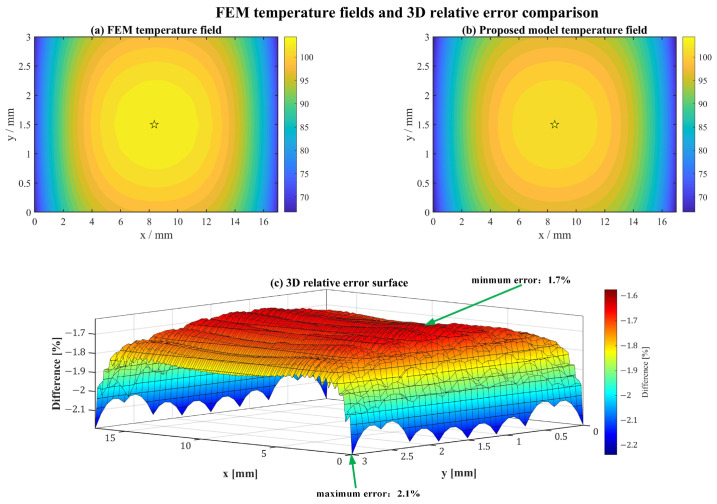
Temperature-distribution comparison between the proposed hybrid model and finite-element simulation.

To verify the temperature-prediction accuracy of the hybrid model, the calculation results of the proposed model are compared with finite-element simulation results under the normal healthy operating condition. The model-validation comparison is shown in Figure 4. The white star in the figure represents the hotspot location.

The temperature-distribution curves along the Y-axis show that the calculation curve of the proposed model is highly consistent with the finite-element simulation results. The predicted maximum internal hotspot temperature of the coil is approximately 116 °C, and the boundary temperatures at both ends are approximately 88 °C and 92 °C, which are basically consistent with the finite-element results.

The quantitative comparison results show that the maximum temperature error between the hybrid model and the finite-element simulation is approximately 2.1%. This result preliminarily indicates that the proposed model can closely approximate the finite-element temperature distribution while maintaining low computational complexity.

## 3. Coil Fault Identification Based on the Hybrid Thermal Model and Neural Network

The long-term reliability of relay coils depends largely on the health state of the insulating varnish film. Under long-term coupled electrical and thermal stresses, the insulating varnish film undergoes physicochemical degradation, local cracking, delamination, and ultimately breakdown. These defects alter the local equivalent thermal resistance or heat-generation characteristics of the coil and macroscopically appear as an elevation, shift, or spike in the temperature-distribution curve. Therefore, the hybrid thermal model established above can be used to generate physically constrained spatial temperature sequences, which are then combined with neural-network-based pattern recognition to enable rapid and non-invasive diagnosis of the coil insulation state.

### 3.1. Electrothermal Coupling-Based Fault Modeling and Spatial Feature Representation

Based on the degradation mechanism of insulation varnish, three typical coil faults with distinct thermodynamic characteristics are selected as recognition targets in this study. Fault-related physical parameters are injected into the established hybrid model combining a composite thermal network and a two-dimensional analytical model. This strategy reduces the time cost of generating large-scale samples using conventional finite element simulations and enables rapid construction of fault temperature-field datasets under multiple operating conditions.

The first type of fault is thermal aging and electrical degradation. Long-term high-temperature operation causes thermal oxidation and molecular chain scission in the varnish layer, leading to material embrittlement and an increase in microvoids. Macroscopically, this fault is represented by a decrease in the equivalent thermal conductivity (*λ_x_*, *λ_y_*), which causes an overall variation in the cross-sectional temperature gradient and an increase in the central hotspot temperature, while the two-dimensional temperature distribution remains approximately symmetric. The second type of fault is varnish cracking and local delamination. Frequent electrothermal cycling and thermal expansion mismatch may induce microcracks or peeling in the insulation layer, and the resulting air gaps form local high-thermal-resistance regions. In the composite thermal-network topology, this fault is characterized by an increase in the conductive thermal resistance of specific directional branches, causing asymmetric distortion of the temperature curve on the damaged side and a shift in the hotspot toward the high-resistance region. The third type of fault is local inter-turn short circuit caused by the development of electrical treeing. Carbonized channels formed by partial-discharge erosion may lead to insulation breakdown, and the short-circuit circulating current generates additional Joule heat. In the two-dimensional analytical model, this effect can be equivalently represented as an additional heat source term (Δ*q_v_*) within a local region, resulting in local temperature peaks and significant gradient variations.

Through the above parameterized injection strategy, different fault mechanisms can be mapped to three categories of spatial thermal features: degradation of thermal conductivity, local distortion of thermal resistance, and enhancement of additional heat sources. This method preserves the physical interpretability of fault evolution while rapidly generating temperature-field samples with differentiated spatial distribution characteristics, thereby providing a data basis for subsequent fault recognition model training and feature dimensionality-reduction analysis.

For the three typical coil faults, the following parameter-injection mechanisms are designed in this study.

1. Simulation of thermal aging: Attenuation of the equivalent thermal conductivity.

Thermal aging causes embrittlement of the insulating varnish film and an increase in microporosity, which macroscopically results in reduced overall thermal conductivity of the coil. An aging attenuation coefficient *k_a_*∈(0, 1] is introduced into the Poisson equation of the two-dimensional analytical model, and the equivalent thermal conductivities (*λ_x_*, *λ_y_*) in the healthy state are modified as follows:

(10) $$\left\{\begin{array}{l} \lambda_{x^{\prime }}=k_{a}\cdot \lambda_{x}\;\\ \lambda_{y^{\prime }}=k_{a}\cdot \lambda_{y} \end{array}\right.$$

During data generation, Monte Carlo random sampling can be performed within *k_a_*∈[0.5, 1.0] to simulate a continuous degradation process from the healthy state to different degrees of aging.

2. Simulation of inter-turn short-circuit: Spatial lattice injection of an inhomogeneous heat source.

An inter-turn short circuit generates abnormally concentrated Joule heat at the insulation-damage location. In this study, the constant internal heat source *q_v_* is modified into a spatial step function *q_v_*(*x*,*y*) containing the fault location (*x_f_*,*y_f_*). Assuming that the short-circuit region is a small rectangular domain *Ω_f_*, the heat-source distribution is defined as follows:

(11) $$q_{v}(x,y)=\left\{\begin{array}{ll} q_{v}+\Delta q_{v}, & \left(x,y)\in \Omega_{f}(x_{f},y_{f}\right)\\ q_{v}, & \left(x,y)\notin \Omega_{f}(x_{f},y_{f}\right) \end{array}\right.$$

Here, Δ*q_v_* denotes the increment in abnormal heat-generation rate caused by the short circuit. By grid-based traversal of different (*x_f_*,*y_f_*) coordinates within the W × H cross-section and random assignment of Δ*q_v_* values with different intensities, the Fourier coefficients of the particular solution term in the analytical model are changed accordingly, thereby reproducing local temperature spikes.

3. Simulation of varnish-film delamination: Asymmetric perturbation of the reusable thermal resistance.

Delamination between the varnish film and the conductor or bobbin introduces high-thermal-resistance air gaps. Because the reusable thermal resistance Rm in the composite thermal circuit directly governs heat transfer in the boundary direction, this study simulates local delamination by injecting a fault multiplier *k_b_* (*k_b_* > 1) into the reusable thermal resistance in a specified direction:

If delamination on the inner surface (core side) is simulated, the reusable thermal resistance of the left branch is modified as follows:

(12) $$R_{L^{\prime }}=k_{b}\cdot R_{m}$$

If end-face delamination is simulated, the thermal resistance of the upper or lower branch is modified.

This parameter injection disrupts the approximate symmetry of the basic composite thermal circuit, deflects the boundary temperature conditions, and drives the two-dimensional analytical model to generate temperature-distribution curves with overall offset and asymmetric distortion.

4. Composite-fault extension analysis.

Although the present classifier is trained for three single-fault categories, the physical parameter-injection framework can be extended to coupled relay coil faults by superimposing multiple degradation mechanisms in the hybrid thermal model. For example, when thermal aging, local inter-turn short circuit, and varnish-film delamination coexist, the equivalent thermal conductivities, internal heat source, and directional reusable thermal resistance can be jointly modified as follows:

(13) $$\left\{\begin{array}{l} \lambda_{x}^{*}=k_{a}\cdot \lambda_{x}\\ \lambda_{y}^{*}=k_{a}\cdot \lambda_{y}\\ q_{v}^{*}(x,y)=q_{v}+\Delta q_{v},\;\;\;\;(x,y)\in \Omega_{f}(x_{f},y_{f})\\ q_{v}^{*}(x,y)=q_{v},\;\;\;\;(x,y)\notin \Omega_{f}(x_{f},y_{f})\\ R_{L^{\prime }}^{*}=k_{b}\cdot R_{m} \end{array}\right.$$

where the superscript * denotes the equivalent parameter under the compound-fault condition; the first two equations describe thermal-conductivity attenuation caused by aging, the third and fourth equations describe the local short-circuit heat-source region and non-fault region separately, and the last equation describes the branch thermal-resistance increase caused by delamination. This format preserves the original three single-fault mechanisms while avoiding a new symbol system.

This formulation indicates that composite faults produce mixed spatial features, including overall temperature elevation, local temperature spikes, and asymmetric curve distortion. Therefore, the proposed model can provide a mechanism-based basis for future multi-label or hierarchical diagnosis of coupled faults, although the current experimental classification task is limited to single-fault identification.

The parameter ranges used for dataset generation were selected as equivalent physical degradation ranges. Thermal aging was represented by *k_a_* = 0.5–1.0, corresponding in the generated dataset to *λ_x_* = 0.1756–0.3484 W/(m·K) and *λ_y_* = 0.6022–1.1946 W/(m·K), which reflects the reduced effective heat-transfer capability of aged enamel insulation materials. The inter-turn short-circuit fault used a local additional heat-source intensity of 8–30 times the normal volumetric heat-generation rate within a local region of 0.04–0.12 W by 0.10–0.30 H, representing concentrated Joule heating caused by a local low-resistance branch. The enamel-film delamination/local thermal-resistance fault used kb = 3–10 to describe the several-fold increase in local thermal resistance caused by insulation separation, air gaps or poor local contact.

### 3.2. Dimensionality Reduction and Vectorization of Temperature Features Based on Orthogonal Symmetry Axes

If the complete two-dimensional temperature field, such as a 100 × 100 discrete grid, is directly used as the neural-network input, the feature dimension reaches 10,000, which can easily increase the number of network parameters and the risk of overfitting.

Considering that hotspot shift, overall temperature-rise elevation, and local spikes in the rectangular coil cross-section can be sufficiently reflected along the central horizontal and vertical axes, this study adopts a physics-guided spatial feature dimensionality-reduction strategy. It should be noted that this dimensionality-reduction method is an approximate representation and is applicable to operating conditions in which the fault-induced thermal spot can affect the temperature curves along the central axes.

To reduce the dimensionality of the reconstructed two-dimensional temperature field, the temperature sequences along two orthogonal centerlines are extracted as compact diagnostic features. This strategy converts the spatial temperature distribution into a low-dimensional sequence representation, which decreases the number of input features and improves the computational efficiency of the subsequent 1D-CNN classifier. For the 17 mm × 3 mm relay micro-coil considered in this study, the compact geometry and short thermal diffusion path allow most fault-induced temperature disturbances to be reflected in the global temperature distribution and projected onto the orthogonal centerline sequences.

Nevertheless, this dimensionality-reduction strategy also introduces a spatial–sensitivity trade-off. Since the extracted features are mainly sampled along the two central axes, very weak local thermal anomalies located far from the centerlines, especially early-stage hotspots near edge or corner regions, may be less directly represented than in full-field or dense regional sampling methods. Therefore, the proposed orthogonal centerline feature is more suitable for capturing dominant global thermal distortion and computationally efficient fault classification, while its sensitivity to extremely weak off-axis local anomalies is relatively limited. This limitation is considered in the interpretation of the classification results in Section 4.3.

Specifically, the continuous temperature-distribution sequences along the horizontal centerline (*y* = H/2) and the vertical centerline (*x* = W/2) of the rectangular cross-section are extracted separately. Each line segment is uniformly sampled at *N* points to obtain two one-dimensional sequences, which are then combined into a one-dimensional spatial feature vector **T**_s_:

(14) $$T_{X}=\left[T(x_{1},H/2),T(x_{2},H/2),\ldots ,T(x_{N},H/2)\right]$$

(15) $$T_{Y}=\left[T(W/2,y_{1}),T(W/2,y_{2}),\ldots ,T(W/2,y_{N})\right]$$

(16) $$T_{s}={\left[T_{X},T_{Y}\right]}^{T}$$

**Figure 5:**
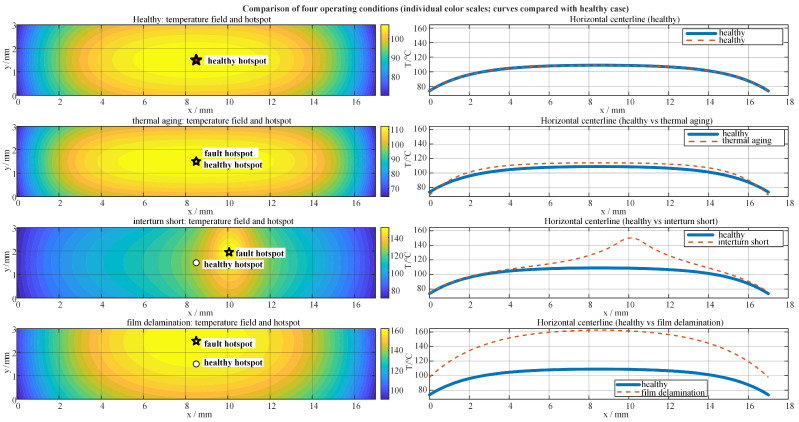
Spatial temperature-sequence characteristics under different relay coil states.

The effects of different coil states on the internal temperature field of the coil are shown in Figure 5. In the normal state, the temperature curve is approximately symmetric and the overall temperature rise is relatively low. Thermal aging reduces the thermal conductivity of the insulating material, causing an overall upward shift in the curve while maintaining approximate symmetry. Local varnish-film delamination impedes heat dissipation on one side, resulting in curve inclination accompanied by hotspot displacement. Inter-turn short circuit, by contrast, produces spikes due to local abnormal heat generation. These differences indicate that the hybrid thermal model can map different coil faults into separable spatial temperature sequences, providing a feature basis for subsequent 1D-CNN identification.

### 3.3. Topology and Training Procedure of the 1D-CNN Fault-Diagnosis Network

**Figure 6:**
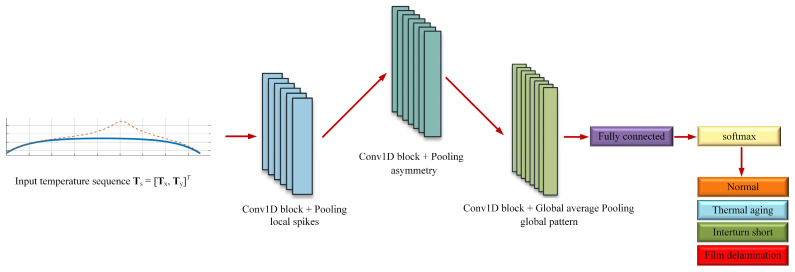
Topology of the proposed 1D-CNN fault-diagnosis network.

To map the dimension-reduced spatial temperature sequence **T**_s_ to specific coil health states, this study employs a one-dimensional convolutional neural network (1D-CNN) as the classifier. The diagnostic network consists of a feature-extraction backbone and a classification-mapping head, with the hierarchical design described as follows. The overall 1D-CNN diagnostic architecture is shown in Figure 6.

The input layer receives the 1 × 2 N-dimensional temperature feature vector generated in Section 3.2. To reduce the influence of dimensional differences on training while preserving the baseline temperature-rise information caused by thermal aging, global Min–Max normalization is adopted in this study.

The convolutional layers are used to extract local and global temperature-curve features. Shallow convolutional layers can capture local spike-like discontinuities caused by inter-turn short circuits, whereas deeper convolutional layers have larger receptive fields and can characterize curve asymmetry and hotspot displacement caused by varnish-film delamination.

The pooling layers downsample the feature maps, reducing the number of parameters while retaining significant temperature-rise distortion features. At the end of feature extraction, global average pooling is introduced instead of the conventional flattening layer to reduce the risk of overfitting.

The fully connected layers perform nonlinear combination and dimensionality reduction in the convolutional features. The output layer contains the same number of neurons as the number of fault classes, and the predicted probability of each health state is obtained using the softmax function.

After determining the 1D-CNN topology, the training quality of the model determines its diagnostic performance. This study adopts a training procedure consisting of multiclass cross-entropy loss, the Adam optimizer, and an early-stopping strategy to learn the nonlinear mapping from the temperature sequence **T**_s_ to the fault-state labels.

(1) A fault sample set is batch-generated using the hybrid thermal model to construct the original feature space.

Here, *y*(*i*) denotes the one-hot encoded label vector corresponding to the coil health state. To ensure the objectivity of model evaluation, stratified random sampling is used to divide the dataset into training, validation, and test sets at proportions of 70%, 15%, and 15%, respectively, ensuring consistent class distributions across all subsets. In each iteration, the training set is further divided into mini-batches of size M to balance computational efficiency and gradient-estimation stability. If multiple neighboring samples are generated from the same physical operating condition through parameter perturbation, the dataset should be split by operating-condition groups to reduce the risk of similar-sample leakage between the training and test sets.

(2) The network performs forward inference for each mini-batch. After mapping through the convolutional, pooling, and fully connected layers, the Softmax layer outputs the predicted probability for each class. Because insulation-state evaluation is a multiclass classification task, multiclass cross-entropy is adopted as the optimization objective:

(17) $$L_{CCE}=-\frac{1}{M}\sum_{i=1}^{M}\sum_{c=1}^{C}y_{i,c}\log\left({\hat{y}}_{i,c}\right)$$

where *M* denotes the mini-batch size and *C* is the total number of fault categories. *y_i_*_,*c*_ is the one-hot encoded ground-truth indicator, where *y_i_*_,*c*_ = 1 if the *i*-th sample belongs to class *c*, and *y_i_*_,*c*_ = 0 otherwise. This loss function optimizes the classification model by measuring the discrepancy between the ground-truth label distribution and the predicted probability distribution. When the predicted probability corresponding to the true class is low, the loss value increases, thereby encouraging the network to improve its discriminative confidence for the correct fault category.

(3) After calculating the batch loss, the gradients of the loss function with respect to all trainable parameters are obtained using the backpropagation algorithm. To improve convergence efficiency on complex high-dimensional loss surfaces, the Adam optimization algorithm is employed. This algorithm simultaneously computes the first- and second-moment estimates of the gradients and adaptively assigns learning rates to different weight parameters:

(18) $$\theta_{t}=\theta_{t-1}-\frac{\eta }{\sqrt{{\hat{v}}_{t}}+\epsilon }{\hat{m}}_{t}$$

where *t* denotes the iteration step; *θ_t_* and *θ_t_*_−1_ represent the model parameters after and before the update at the *t*-th iteration, respectively; *η* is the learning rate; ${\hat{m}}_{t}$ and ${\hat{v}}_{t}$ denote the bias-corrected first-order and second-order moment estimates, respectively; and *ϵ* is a small constant introduced to avoid division by zero. The Adam algorithm adaptively adjusts the update step size for different parameters, thereby improving the model’s ability to capture subtle temperature-gradient distortion features in the ***T_s_*** sequences.

(4) To alleviate overfitting during training on simulated samples, an early-stopping mechanism is introduced. At the end of each training epoch, the validation-set loss is evaluated. If the validation loss no longer decreases within the specified patience epochs, training is terminated and the weights with the best validation performance are saved. During online diagnosis, the spatial temperature sequence reconstructed from real-time boundary conditions is input into the 1D-CNN. The Softmax layer outputs the probabilities of each class, and the class with the maximum probability is taken as the diagnostic result, with the corresponding probability serving as the diagnostic confidence.

**Figure 7:**
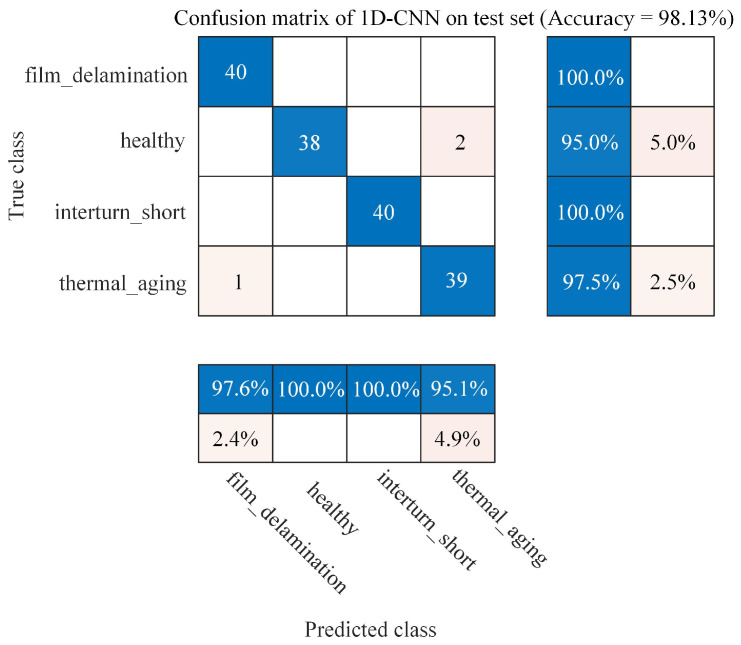
Confusion matrix of the 1D-CNN classification results on the test samples.

To evaluate the recognition performance of the 1D-CNN, 160 test samples not involved in training are used for final testing, and the classification results are presented using a confusion matrix. The corresponding classification result is shown in Figure 7.

As shown in Figure 7, the proposed method achieves an overall classification accuracy of 98.13%, indicating that the orthogonal centerline temperature sequences retain the main discriminative information for the four relay coil states. The high accuracy can be attributed to the compact geometry of the 17 mm × 3 mm micro-coil, in which the short thermal diffusion distance enables local heat-source variation or insulation degradation to affect the overall temperature field and, consequently, the two centerline sequences.

However, the few misclassified samples in the confusion matrix also reflect the geometric sensitivity limitation of the centerline-based feature representation. For neighboring states with relatively weak thermal differences, such as normal and slight aging conditions, the temperature-field variation may be small and spatially dispersed. If the corresponding local thermal disturbance is located away from the two centerlines, its projection onto the extracted orthogonal sequences becomes less distinctive, which may reduce the separability between adjacent classes. Therefore, these occasional misclassifications are consistent with the spatial–sampling trade-off discussed in Section 3.2: the centerline strategy improves feature compactness and computational efficiency, but may slightly weaken the representation of very small off-axis local anomalies.

In the confusion matrix, the vertical axis represents the true fault type, and the horizontal axis represents the model-predicted type. The diagonal elements indicate the number of correctly classified samples.

The test results show that, among the 160 test samples, the model correctly identifies 157 samples, yielding an overall accuracy of 98.13%. Specifically, two normal-state samples are misclassified as mild aging, and one mild-aging sample is misclassified as local delamination. These confusions mainly occur between early degradation states with weak temperature variations and similar curve morphologies, which is consistent with the physical fact that thermal-feature differences are small in the early stage of insulation aging. For more destructive faults such as local delamination and inter-turn short circuit, no missed detections occur in the test set.

It should be noted that the present 1D-CNN has not yet been optimized by pruning, quantization, or other lightweight strategies. In the current diagnostic workflow, the on-site device mainly acquires the relay temperature sequence and transmits the feature data to a host/cloud platform for inference, rather than executing the complete network directly on a low-power embedded terminal. Direct embedded deployment for fully local real-time diagnosis will be further investigated in future work through lightweight optimization strategies such as structured pruning, low-bit quantization, knowledge distillation, and edge–cloud collaborative inference.

## 4. Experimental Validation and Steady-State Boundary-Driven Model Prediction

### 4.1. Experimental Platform and Measurement-Point Arrangement

**Figure 8:**
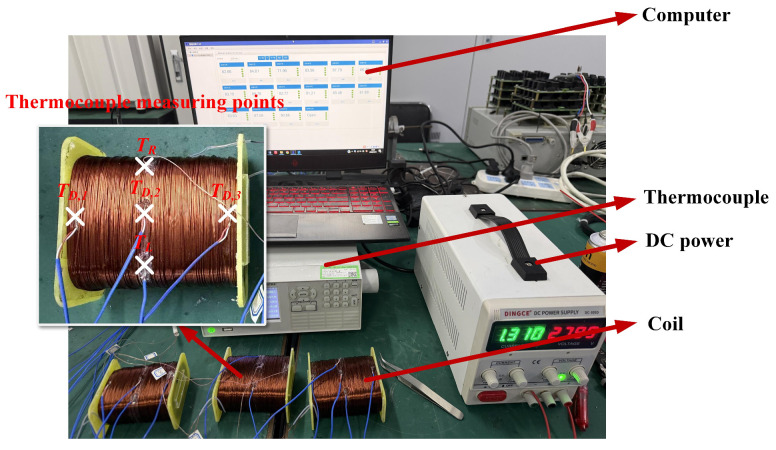
Coil temperature-monitoring experimental platform used for steady-state boundary measurement.

To verify the applicability of the proposed composite thermal-circuit–two-dimensional analytical coupling model under real operating conditions, a coil temperature-monitoring experimental platform was established, as shown in Figure 8. To facilitate thermocouple placement and fault-state simulation, the experimental object in this study was a 5.6-times enlarged substitute model of the original relay coil. According to the fabrication drawings, the rectangular computational region used for two-dimensional analytical reconstruction in the enlarged model had a width of W = 40.56 mm and a height of H = 14.75 mm. A regulated adjustable DC power supply was used to provide constant-power excitation, and boundary temperature data were recorded after the system reached steady state.

**Figure 9:**
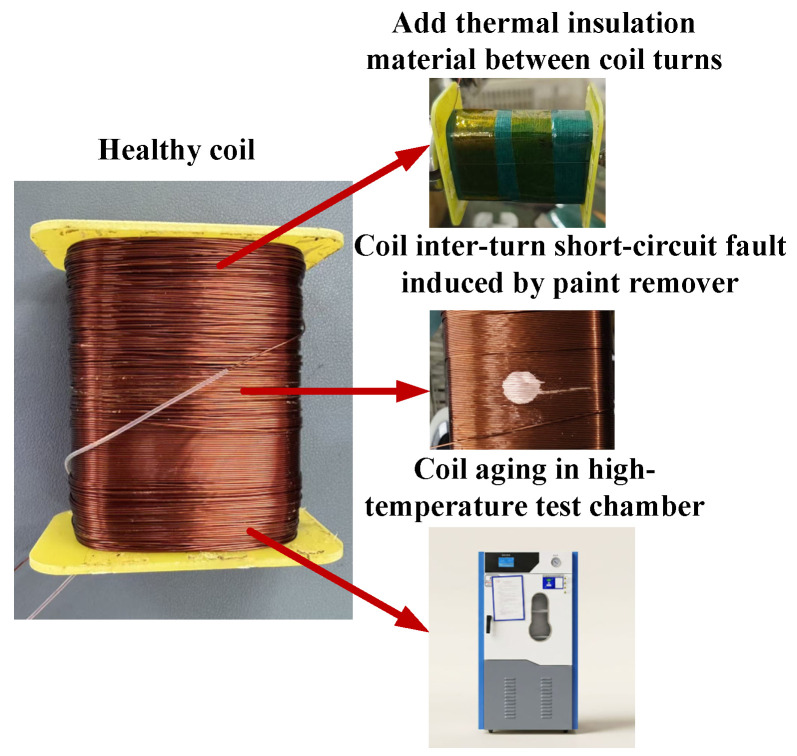
Relay coil samples prepared to simulate local inter-turn short-circuit faults.

The experimental coils included two healthy coils, two coils simulating inter-turn short-circuits, two coils simulating enamel-film delamination, and two coils simulating thermal aging. Inter-turn short-circuits were simulated by treating a local enamel-film region with paint remover to form a low-insulation area. Enamel-film delamination was simulated by introducing thermal insulation material during coil winding to form a local high-thermal-resistance region. Thermal aging was obtained by placing the coils in a 125 °C temperature chamber for 24 h and 48 h, respectively. Representative healthy coil samples and simulated fault samples are shown in Figure 9.

The experimental characterization further supports these equivalent ranges. Thermal aging increased the overall temperature rise while the mean coil resistance changed by only −0.27%, indicating that the ageing treatment mainly affected thermal rather than electrical parameters. The mean resistance of the inter-turn short-circuit coils decreased by 2.76%, supporting the low-resistance-branch assumption. For the local thermal-resistance fault, representative temperature differences of 1.30 °C and 2.26 °C were observed across the local insulating region, confirming the local thermal-barrier effect.

**Figure 10:**
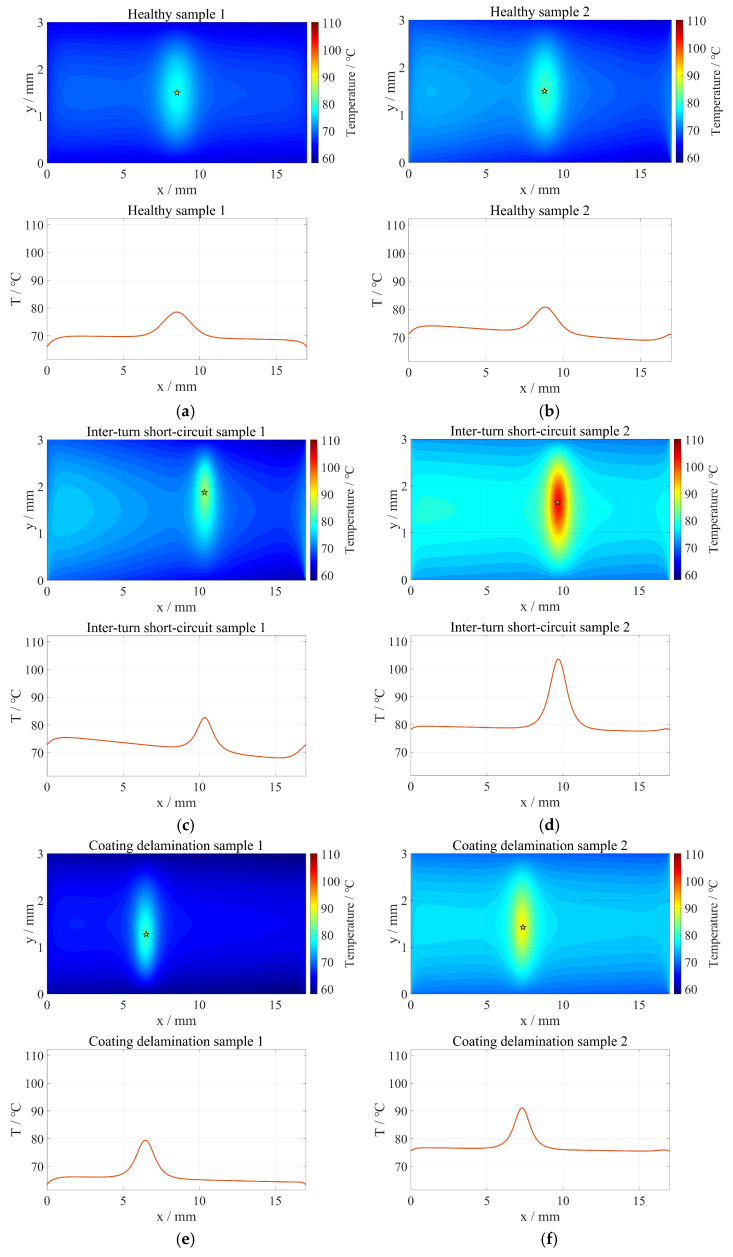

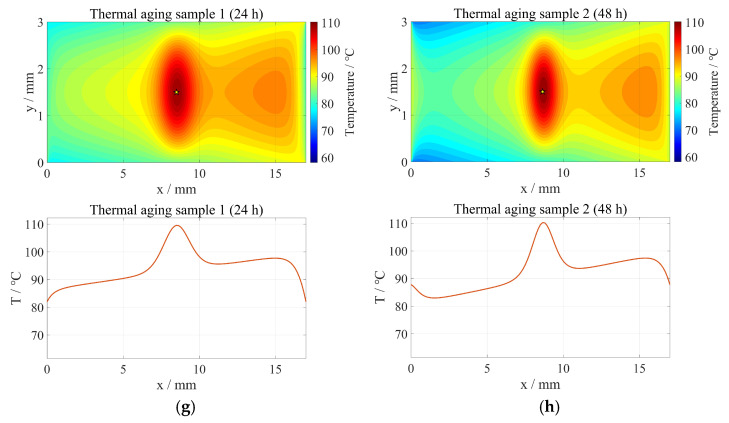
Hotspot temperature-field distributions of eight relay coil samples under different operating conditions: (**a**) healthy sample 1; (**b**) healthy sample 2; (**c**) inter-turn short-circuit sample 1; (**d**) inter-turn short-circuit sample 2; (**e**) coating delamination sample 1; (**f**) coating delamination sample 2; (**g**) thermal aging sample after 24 h; and (**h**) thermal aging sample after 48 h.

Figure 10 presents the hotspot temperature-field distributions of eight relay coil samples under different operating conditions, including healthy, inter-turn short-circuit, coating delamination, and thermal aging states. Each subfigure corresponds to an independent experimental sample, and the color variation reflects the reconstructed temperature distribution within the coil cross-section. The red and yellow regions indicate high-temperature hotspot areas, whereas the blue regions represent relatively low-temperature regions. Additionally, the white star indicates the hotspot location, and the red line represents the temperature curve. Compared with the healthy samples, the faulty samples exhibit distinguishable differences in hotspot location, hotspot intensity, and temperature-gradient distribution. In particular, the inter-turn short-circuit samples show more localized high-temperature regions, while the coating delamination and thermal aging samples present altered thermal diffusion characteristics caused by insulation degradation or heat-transfer deterioration. These differences demonstrate that the experimentally measured boundary conditions can produce distinguishable spatial thermal-field features, providing a visual basis for subsequent feature extraction and fault-identification analysis.

The 5.6-times enlarged prototype should be interpreted as a proof-of-concept platform rather than a thermo-physical equivalent of the practical miniature relay coil. With geometric enlargement, the thermal mass increases faster than the external heat-dissipation area because the volume scales approximately with *k_s_*^3^ whereas the area scales approximately with *k_s_*^2^. Therefore, the enlarged prototype has a larger thermal-mass-to-area ratio and a longer apparent thermal time constant, which explains the slower boundary-temperature variation observed during repeated energization tests.

The enlargement also changes the convective heat-transfer coefficient, boundary condition, winding geometry, and local fault representation. In particular, the practical relay coil is constrained by the bobbin, iron-core side, relay housing, and compact internal air space, whereas the enlarged prototype has different exposed surfaces and contact conditions. The enlarged winding also changes the relative inter-turn spacing, contact state, and local heat-spreading path, and the simulated inter-turn short-circuit, enamel-film delamination, and thermal aging states reproduce representative thermal disturbance mechanisms rather than exact microscopic defect geometries.

For these reasons, the enlarged experiment is not used for direct absolute-temperature mapping to practical miniature relays. Instead, the measured surface temperatures are imposed as Dirichlet boundary conditions, namely *T_L_*, *T_R_*, *T_D_*, and *T_U_*, so that the reconstruction is driven by the measured boundary thermal state rather than by an assumed scaled heat-transfer coefficient. Thus, the enlarged experiment mainly validates the boundary-driven thermal-field reconstruction mechanism, while the applicability to the miniature-coil geometry is supported by the simulation-based validation and finite-element comparison.

### 4.2. Hybrid Thermal-Model Reconstruction Driven by Steady-State Boundary Conditions

To bridge the spatial-topology difference between the three-dimensional physical experimental environment and the two-dimensional analytical mathematical model, this paper proposes a dimensionality-reduction mapping mechanism based on feature-point substitution and spatial averaging. Because an actual relay coil has a finite axial length, end-face heat dissipation and nonuniform local cooling conditions cause the temperature-rise distribution to exhibit complex three-dimensional characteristics. Therefore, before driving the two-dimensional model calculation, the five discrete temperature-measurement points distributed on the coil surface must be effectively mapped into the four ideal boundary conditions required by the model, namely *T_L_*, *T_R_*, *T_D_*, and *T_U_*.

The specific equivalent mapping logic is as follows:

1. Spatial averaging treatment of the lower boundary *T_D_*.

The lower-boundary temperature is no longer represented by a single measurement point, but is instead taken as the spatial average of three feature points located along the bottom centerline. These three measurement points are located at the bottom of the central cross-section and at the edge positions of the two axial ends. By introducing the spatial weighting coefficient *ω_i_*, discrete temperatures with axial differences can be converted into the equivalent steady-state boundary value required for the two-dimensional cross-section. The expression is given as follows:

(19) $$T_{D}=\sum_{i=1}^{3}\omega_{i}T_{D,i}$$

where *T_D_*_,*i*_ denotes the steady-state temperature of the i-th measurement point on the bottom centerline. Considering the approximate consistency of the heat-dissipation environment, an equal-weight averaging strategy is adopted in this study (ωi = 1/3). This treatment effectively suppresses the local disturbance of boundary heat flux caused by three-dimensional end-face heat dissipation and provides a boundary input with greater global representativeness.

2. Feature-point mapping of the left and right boundaries *T_L_* and *T_R_*.

For the left and right boundaries of the analytical model, the temperatures at the left corner point *T*(0,0) and right corner point *T*(W,0) at the bottom of the central cross-section are directly extracted as equivalent inputs with reference to the coordinate system shown in Figure 1. This mapping scheme can directly capture the dominant temperature-gradient characteristics of heat dissipation from the central cross-section toward the iron-core side, i.e., the radial inner side, and toward the ambient side, i.e., the radial outer side. It also minimizes the difficulty of arranging experimental measurement points while ensuring that key heat-generation features are not lost.

3. Temperature-rise proportional correction of the upper boundary *T_U_*.

Considering the slight differences in heat-dissipation paths and contact conditions between the upper and lower end faces of the coil, the upper-boundary temperature is corrected proportionally based on the lower-boundary temperature rise, so as to reflect the non-absolutely symmetric thermal-response relationship between the two boundaries. The correction formula is defined as follows:

(20) $$T_{U}=T_{0}+\eta_{U}\left(T_{D}-T_{0}\right)$$

where *T*_0_ is the ambient reference temperature, taken as 25 °C, and *η_U_* is the upper-boundary temperature-rise correction coefficient. According to the calibration results of the boundary response of the hybrid model, *η_U_* = 0.95 is adopted in this study to describe the slightly lower equivalent temperature-rise level of the upper boundary relative to the lower boundary. The schematic diagram of the five thermocouple measurement points, *T_D_*_,1_, *T_D_*_,2_, *T_D_*_,3_, *T_R_*, and *T_L_*, is shown in Figure 9. Table 4 presents the boundary conditions corresponding to the experimentally measured values of the eight samples.

### 4.3. Fault-Identification Results and Discriminant Analysis

**Figure 11:**
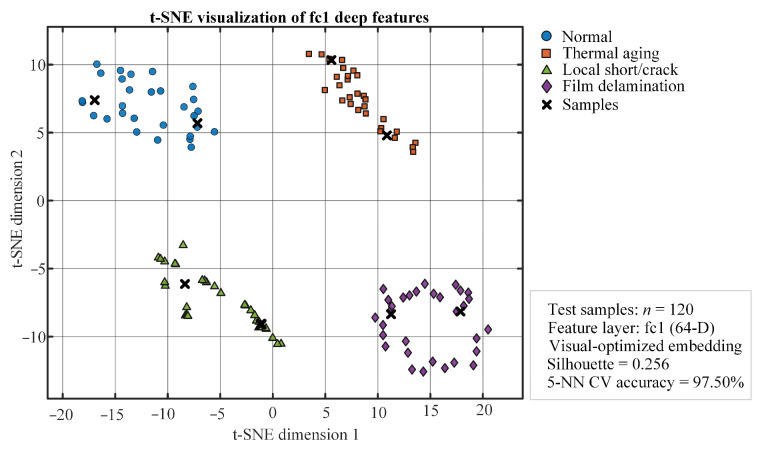
t-SNE visualization of the 64-dimensional deep features extracted from the fc1 layer.

To further demonstrate the feature-extraction capability of the 1D-CNN for temperature sequences corresponding to different coil states, the 64-dimensional deep features output by the fully connected layer fc1 of the trained network were extracted and reduced to a two-dimensional space using t-SNE for visualization, as shown in Figure 11. Different colors and symbols in the figure denote the normal state, thermal aging, inter-turn short-circuit, and enamel-film delamination, respectively, while the black crosses represent the reference centers of the subclusters within each class. The four types of samples form relatively clear regional distributions in the two-dimensional feature space, indicating that the network can learn discriminative features related to coil states from the reconstructed temperature sequences.

As shown in Figure 11, the deep features extracted from the fully connected layer fc1 of the 1D-CNN model exhibit good discriminative capability for different coil conditions. The samples corresponding to normal condition, thermal aging, local short/crack, and film delamination form relatively independent clusters in the two-dimensional feature space, indicating that the model learns not only the overall variation trend of the temperature sequences but also the differences in thermal response characteristics under different fault states. In particular, the film delamination samples are mainly distributed in the lower-right region with a clear boundary from the other conditions. Although the normal and thermal aging samples show a certain degree of dispersion, their overall feature distributions remain distinguishable. Combined with the high recognition accuracy on the test set, these results demonstrate that the proposed 1D-CNN model has effective feature e-traction capability and satisfactory fault-identification performance for different fault states of the relay coil.

## 5. Conclusions

Taking the micro coil of relays with forcibly guided contacts as a representative research object, this paper proposes a novel fault-identification method for micro coils in electromechanical elementary components, integrating a composite thermal circuit, a two-dimensional analytical thermal model, and a one-dimensional convolutional neural network (1D-CNN). By iteratively correcting reusable thermal resistances, the proposed hybrid thermal model achieves accurate two-dimensional temperature-field representation at a low computational cost, yielding a temperature error of approximately 1.7% compared to finite-element analysis results. To simulate thermal aging, enamel-film delamination, and localized inter-turn short circuits, corresponding physical parameter-injection mechanisms are established to batch-generate training data. Based on the one-dimensional spatial temperature features extracted from the cross-sectional orthogonal central axes, the constructed 1D-CNN model achieves an overall recognition accuracy of 98.13%. By arranging identical temperature measuring points, this method can effectively learn and extract fault-discriminative features from spatial temperature sequences, providing a novel technical route characterized by both high accuracy and physical mechanism constraints for the early fault diagnosis and insulation-state assessment of micro coils in electromechanical elementary components. Although the proposed method verifies the feasibility of thermal-model-based relay coil fault diagnosis, several limitations remain. The experimental dataset is still limited, and the enlarged coil model was mainly used to obtain clearer and repeatable thermal responses for mechanism verification. In addition, the present thermal analysis is based on a steady-state assumption and does not fully represent transient heating and cooling during practical relay operation. Future work will further evaluate the applicability and robustness of the proposed method under more engineering-relevant operating conditions and broader validation scenarios.

## Figures and Tables

**Table 1:** Comparison between previous studies and this work.

Study Type	Object	Method	Validation	Difference
Contact studies [1,4]	Contact pairs	Spot analysis Resistance prediction	Measured data	Contact focused
Relay models [2,3]	Contact heat Magnetic material	Heat-source model Material evaluation	Simulation tests	No coil diagnosis
Motor winding diagnosis [11,23]	Motor windings	Thermal model CNN diagnosis	FEM validation Experimental data	Different geometry
This work	Relay micro-coils	Thermal reconstruction 1D-CNN diagnosis	FEM validation Enlarged-coil tests	Coil fault diagnosis

**Table 2:** Main input parameters of the hybrid model.

Symbol	Parameters	Value
*W*	Width of computational region	17 mm
*H*	Height of computational region	3 mm
*λ_x_*	Thermal conductivity in the *x* direction	0.35 W/(m·K)
*λ_y_*	Thermal conductivity in the *y* direction	1.20 W/(m·K)
*q_v_*	Volumetric heat-generation rate	6.0 × 10^6^ W/m^3^
*T* *_a_*	Ambient temperature	25 °C
*R_m,_*_0_	Initial boundary thermal resistance	[0.0102, 0.0069, 0.0094, 0.0071] K/W

**Table 3:** Normalized sensitivity coefficients of key thermal parameters.

Parameter	Perturbation	*|S* *_T_avg_|*	*|S* *_T_max_|*	*|S* *_s_eq_|*
*λ_x_*	±10%	0.018	0.021	0.032
*λ_y_*	±10%	0.033	0.054	0.048
*q_v_*	±10%	0.748	0.771	0.758
*h*	±20%	0.726	0.724	0.720
*R_m_*	±10%	0.697	0.696	0.692
*T_b_*	±1 °C	0.252	0.229	0.243

**Table 4:** Boundary conditions corresponding to the experimentally measured values of the eight coil samples.

Sample No.	State Description	*T_L_*/°C	*T_R_*/°C	*T_D_*/°C	*T_U_*/°C
Sample 1	Healthy 1	64.08	62.41	66.17	64.11
Sample 2	Healthy 2	68.86	62.11	71.60	69.27
Sample 3	Inter-turn short-circuit 1	70.38	60.71	73.25	70.84
Sample 4	Inter-turn short-circuit 2	73.45	71.19	78.51	75.83
Sample 5	Enamel-film delamination fault 1	60.50	57.73	63.26	61.35
Sample 6	Enamel-film delamination fault 2	70.80	68.59	75.28	72.77
Sample 7	Thermal aging for 24 h	77.94	91.25	77.35	74.73
Sample 8	Thermal aging for 48 h	71.15	90.28	81.67	78.83

## Data Availability

The data that support the findings of this study are available from the corresponding author upon reasonable request.
